# Incidence and outcomes of anal and cervical cancer among adults with HIV in Latin America: a retrospective cohort study

**DOI:** 10.1002/jia2.70050

**Published:** 2025-10-11

**Authors:** Rachael A. Pellegrino, Shengxin Tu, Rodrigo Ville‐Benavides, Emilia M. Jalil, Staci L. Sudenga, Brenda Crabtree‐Ramírez, Claudia P. Cortes, Diana Varela, Genevieve Hilaire, Cynthia Riviere, Eduardo Gotuzzo, Bryan E. Shepherd, Valeria Fink, Jessica L. Castilho, the Caribbean, Central and South America network for HIV Epidemiology

**Affiliations:** ^1^ Division of Infectious Diseases Department of Medicine Vanderbilt University Medical Center Nashville Tennessee USA; ^2^ Department of Biostatistics Vanderbilt University Medical Center Nashville Tennessee USA; ^3^ Departamento de Infectología Instituto Nacional de Ciencias Médicas y Nutrición Salvador Zubirán Mexico City Mexico; ^4^ Instituto Nacional de Infectologia Evandro Chagas Rio de Janeiro Brazil; ^5^ Division of Epidemiology Department of Medicine Vanderbilt University Medical Center Nashville Tennessee USA; ^6^ Fundación Arriarán and University of Chile School of Medicine Santiago Chile; ^7^ Servicio de Atención Integral del Hospital Escuela Tegucigalpa Honduras; ^8^ Les Centres GHESKIO Port‐au‐Prince Haïti; ^9^ Universidad Peruana Cayetano Heredia, Instituto de Medicina Tropical Alexander von Humboldt Lima Peru; ^10^ Research Department Fundacion Huesped Buenos Aires Argentina; ^11^ Department of Health Policy Vanderbilt University Medical Center Nashville Tennessee USA

**Keywords:** HIV, anal cancer, cervical cancer, incidence, Latin America, cancer screening

## Abstract

**Introduction:**

Human papillomavirus (HPV)‐associated cervical and anal cancers disproportionately affect people with HIV (PWH). This study aimed to determine the incidence trends of and risk factors for these malignancies in PWH in Latin America.

**Methods:**

We included PWH from the Caribbean, Central and South America network for HIV epidemiology (CCASAnet) who contributed person‐time between 2000 and 2019. We calculated crude and age‐standardized incidence rates, examining trends over time with Poisson regression. Adjusted hazard ratios were calculated using Cox proportional hazard models with propensity score adjustment. We calculated the probability of survival after cancer diagnosis using Kaplan−Meier curves. To understand factors that influence our results, we surveyed all adult CCASAnet sites on current practices of cervical and anal cancer screening.

**Results:**

Overall, 5739 females with HIV (43,417 person‐years) were included in cervical cancer analyses. There were 27 incident cervical cancers: crude incidence rate of 62.2 (95% confidence interval [CI]: 34.9−89.4) per 100,000 person years. In the anal cancer analysis, 12,489 males who have sex with men (MSM), 7324 males other than MSM and 5739 females were included for a total of 25,552 PWH, contributing 157,166 person‐years. Anal cancer was diagnosed in 56 individuals: crude incidence rates of 59.1 [95% CI: 33.2−85.0], 20.7 [95% CI: 11.6−29.7] and 15.2 [95% CI: 8.6−21.9] per 100,000 person‐years in MSM, females and males other than MSM, respectively. Age‐standardized incidence rates did not significantly change over time. Anal cancer risk decreased significantly with higher time‐updated CD4 cell count. The predicted probability of 5‐year survival after cancer diagnosis was 72.6% (95% CI: 48.4−86.8) for cervical cancer and 58.5% (95% CI: 44.0−70.5) for anal cancer.

**Conclusions:**

In one of the few reports outside the United States or Europe, we did not observe a decrease in age‐standardized incidence rates for anal and cervical cancer between 2000 and 2019. These data support continued efforts for cancer prevention through access to gender‐neutral HPV vaccination and cancer screening.

## 1 INTRODUCTION

Human papillomavirus (HPV) is a common sexually transmitted infection associated with nearly all cases of cervical cancer and anal cancer [1, 2]. While HPV may clear spontaneously after acquisition, a small portion will lead to the development of pre‐cancerous squamous intraepithelial lesions, which can progress to invasive cancer [3]. Immunosuppression increases carcinogenicity of persistent HPV, and multiple studies have demonstrated that people with HIV (PWH) not only have higher rates of HPV acquisition and persistence, but also elevated risk of HPV‐associated malignancies compared to individuals without HIV [4, 5, 6]. Furthermore, late presentation and lack of access to effective cancer treatment contribute to high mortality in PWH after cervical cancer diagnosis in many low‐ and middle‐income countries, though there is limited data on anal cancer outcomes in this setting [7, 8].

Standardized cervical cancer screening and treatment of pre‐cancerous lesions have significantly reduced the global incidence of cervical cancer, though regional differences persist [9]. In 2020, the World Health Organization (WHO) adopted the Global Strategy for Cervical Cancer Elimination, which has encouraged renewed efforts to increase access to HPV vaccination, cervical cancer screening and treatment of pre‐cancerous lesions [10]. In contrast, screening for anal cancer is not widely available, and most studies on screening are limited to high‐income countries [4, 11]. This growing body of evidence led to recent consensus guidelines for anal cancer screening, highlighting the importance of continued research to inform implementation of screening and treatment programmes [12, 13].

Monitoring of HPV‐associated cancers in populations at higher risk is especially important in Latin America, where many countries have seen lower than recommended uptake of HPV vaccination and cervical cancer screening [14]. For cervical cancer, estimates of age‐standardized incidence rates in this region remain above the WHO Cervical Cancer Elimination Initiative goal; however, these rates are not specific to PWH, a group that is at higher risk for poor outcomes [15]. For anal cancer, nearly all published studies on incidence and outcomes in PWH are from high‐income countries, creating a gap in data for most of the world, including Latin America [4, 9, 16].

This study aimed to determine the incidence trends of and risk factors for anal and cervical cancers and to evaluate the risk of mortality after an anal or cervical cancer diagnosis in PWH in Latin America using a multinational cohort. Additionally, we described current practices of anal and cervical cancer screening and HPV vaccination at each clinical site to provide context for these results and identify areas for future improvements.

## 2 METHODS

### 2.1 Study design and population

This study included clinical sites from seven Latin American and Caribbean countries included in the Caribbean, Central and South America network for HIV epidemiology (CCASAnet) of the International epidemiology Databases to Evaluate AIDS (IeDEA) consortium [17]. For the analysis of cancer endpoints, we included the six CCASAnet sites that report cancer data: Instituto Nacional de Infectologia Evandro Chagas (Rio de Janeiro, Brazil); Fundación Arriarán (Santiago, Chile); Instituto Hondureño de Seguridad Social and Hospital Escuela Universitario (Tegucigalpa, Honduras); Instituto de Medicina Tropical Alexander von Humboldt, Universidad Peruana Cayetano Heredia (Lima, Peru), Centro Medico Huesped (Buenos Aires, Argentina) and Instituto Nacional de Ciencias Médicas y Nutrición, Salvador Zubirán (Mexico City, Mexico) [17]. These sites are predominantly tertiary referral centres that provide comprehensive HIV care, including preventative care. For the screening survey, Les Centres GHESKIO (Port‐au‐Prince, Haiti) was also included [17]. Clinical data is routinely abstracted from medical charts at each site and sent to the CCASAnet Data Coordinating Center at Vanderbilt University Medical Center, Nashville, TN, USA, for processing and data harmonization. Institutional ethics review boards from all clinical sites and Vanderbilt University approved this study, waiving the requirement for individual patient informed consent.

We excluded all PWH who were <16 years of age at the time of clinic entry and individuals with cervical or anal cancer diagnosed before clinic entry or before 1 January 2000. We included all individuals in care on or after 1 January 2000. Individuals contributed person‐time to the cohort analysis from 1 January 2000, or the date of clinic entry (if after 1 January 2000) through the earliest occurrence of anal or cervical cancer diagnosis, last clinic visit, death or 31 December 2019. Data after 2019 were censored due to disruptions in clinical care and data reporting during the COVID‐19 pandemic. All PWH contributed person‐time in the anal cancer analysis. Only individuals assigned female sex at birth contributed person‐time in the cervical cancer analysis. Data on gender is not consistently reported, and thus, sex assigned at birth was used. Each reported case of anal and cervical cancer was validated through chart review and pathology by clinical staff at sites. We only included incident diagnoses with histologic confirmation or clear documentation of invasive cervical and anal cancer, excluding cases of pre‐cancerous lesions. Data on cervical and anal cancer screening results are not routinely reported from CCASAnet sites.

### 2.2 Incidence rates and temporal trends

We calculated the overall crude incidence of anal and cervical cancer and overall age‐standardized incidence rates using the WHO standard population [18]. To assess trends over time, we calculated the age‐standardized incidence rate for each calendar year and plotted these values. We then built Poisson regression models for cervical cancer and anal cancer using calendar year as the exposure and log‐transformed person‐years as an offset. Age was included as a covariate, as well as sex/sexual HIV acquisition risk group (men who have sex with men [MSM], males other than MSM, females) for anal cancer. The age‐standardized incidence rates were computed as a weighted average of the age‐specific rates, with the weights reflecting the relative age distribution of the WHO standard population. All cases diagnosed after clinic enrolment were included in the incidence analyses. Preplanned sensitivity analyses were performed for each incidence trend that limited cases to those diagnosed >90 days after clinic enrolment, excluding individuals with ≤ 90 days of follow‐up.

### 2.3 Risk factors and mortality

To account for the increased cancer risk with ageing, we used age as a unit of time for the univariate Cox proportional hazards models stratified by clinical site for cervical cancer and anal cancer to assess demographic and clinical predictors of cancer including calendar year of clinic entry, time‐updated CD4 cell count (most recent measurement up to 1 year, at which point, it was assigned as missing), time‐updated antiretroviral therapy (ART) initiation status (ever started ART vs. not yet or never started ART), and, for the anal cancer model, sex/sexual HIV acquisition risk group ascertained at clinic entry (MSM, males other than MSM, females). Once an individual was recorded as having ever started ART, they remained in this group. To avoid the linearity assumption, all the continuous predictors were expanded with restricted cubic splines (each with three knots). For each predictor, we built a propensity‐score‐adjusted Cox proportional hazard model stratified by clinical site and accounting for all other covariates for both cervical cancer and anal cancer. Given the small number of events, these propensity score models were used to avoid overfitting. The propensity score models were linear regression models for continuous variables (time‐updated age, calendar year of clinic entry, time‐updated CD4), logistic regression models for binary variables (time‐updated ART use) and multinomial logistic regression models for categorical variables (sex/sexual HIV acquisition risk group). The propensity score in the Cox model was also expanded with restricted cubic splines (three knots). Missing data were imputed by multiple imputation (20 times).

Lastly, we evaluated the risk of all‐cause mortality after a diagnosis of cervical or anal cancer. We calculated the predicted probability of 5‐year survival for each malignancy using Kaplan−Meier curves. Data were analysed using R version 4.2.1 and Stata 15.

### 2.4 Clinical site cancer screening and HPV vaccination practices

To understand factors that influence HPV‐associated cancer prevention, diagnosis and treatment, we surveyed all adult CCASAnet sites to gather information on current practices of HPV vaccination and cervical and anal cancer screening. The site survey was adapted from a similar survey conducted in 2012. Detailed questions on anal cancer screening methods were added in the 2023 survey. Data were collected and managed using Research Electronic Data Capture (REDCap) web‐based software hosted at Vanderbilt University [19, 20]. A clinician at each site completed the survey reflecting clinical practices and protocols. In 2012, nine clinical sites were surveyed from seven CCASAnet countries. In 2023, seven clinical sites were surveyed, one from each country, all of which were included in the 2012 survey. Descriptive analyses of these surveys were performed.

## 3 RESULTS

### 3.1 Study population

In total, 57,324 PWH were included in the overall CCASAnet cohort from 2000 through 2019. Of these, the 27,326 PWH enrolled in Haiti were excluded, as no cancer outcomes were available. We excluded 4431 PWH who had enrolled after 31 December 2019, were <16 years of age at clinic entry or had died or been diagnosed with anal or cervical cancer before 1 January 2000. Eight individuals with prevalent cervical cancer and seven individuals with prevalent anal cancer prior to enrollment were excluded (Figure S1). Of the 25,552 included in the cohort study, only 1184 (4.6%) entered the clinic before 1 January 2000. In total, 5739 females with HIV (43,417 person‐years) were included in cervical cancer analyses. In the anal cancer analysis, 12,489 MSM, 7324 males other than MSM and 5739 females, for a total of 25,552 PWH, contributed 157,166 person‐years.

Of those included in the cervical cancer analysis, the median CD4 cell count was 269 cells/µl (interquartile range [IQR] 110–475), and 62.4% were ART‐naïve at clinic entry. The median follow‐up time was 6.5 years (IQR 2.2−12.2) and 13.3% died during follow‐up. Most participants were enrolled in Peru and Brazil, and heterosexual contact was the most common HIV acquisition risk factor. Of those included in the anal cancer analysis, the median CD4 cell count was 264 cells/µl (IQR 103–463), and 62.3% were ART‐naïve at clinic entry. The median follow‐up time was 4.5 years (IQR 1.5−10), and 12.2% died during follow‐up. Most participants were enrolled in Peru, Brazil and Chile, and male‐to‐male sexual contact was the most common HIV acquisition risk factor (Table 1). The percentage of those on ART and the median CD4 count of the cohort both increased annually (Figure S2).

**Table 1:** Descriptive characteristics of MSM, males, other than MSM and females with HIV, 2000–2019

	*N*	MSM (*N* = 12,489)	Males, other than MSM (*N* = 7324)	Females (*N* = 5739)	Overall (*N* = 25,552)
Age at clinic entry	25,552				
Median (IQR)		31.0 (25.0−38.0)	36.0 (29.0−45.0)	33.0 (27.0−42.0)	33.0 (27.0−41.0)
Site, *n* (%)	25,552				
Argentina		352 (2.8)	880 (12.0)	416 (7.2)	1648 (6.4)
Brazil		2771 (22.2)	2073 (28.3)	2028 (35.3)	6872 (26.9)
Chile		4247 (34.0)	756 (10.3)	641 (11.2)	5644 (22.1)
Honduras		70 (0.6)	831 (11.3)	612 (10.7)	1513 (5.9)
Mexico		1511 (12.1)	454 (6.2)	235 (4.1)	2200 (8.6)
Peru		3538 (28.3)	2330 (31.8)	1807 (31.5)	7675 (30.0)
Calendar year of clinic entry	25,552				
Median (IQR)		2013 (2008−2017)	2010 (2006−2015)	2009 (2005−2014)	2012 (2006−2016)
ART use at clinic entry: Ever, *n* (%)^a^	23,451	4400 (38.0)	2439 (37.2)	2001 (37.6)	8840 (37.7)
HIV acquisition risk group, *n* (%)^b^	24,626				
Heterosexual		–	4926 (72.1)	4510 (85.0)	9436 (38.3)
MSM		12,489 (100)	–	–	12,489 (50.7)
Other^c^		–	332 (4.9)	282 (5.3)	614 (2.5)
Unknown		–	1574 (23.0)	513 (9.7)	2087 (8.5)
CD4 count at clinic entry (cells/µl)	23,171				
Median (IQR)		304.0 (135.0−489.0)	192.0 (64.0−381.1)	269.0 (110.0−475.0)	264.0 (103.0−463.0)
Follow‐up years since cohort entry	25,552				
Median (IQR)		3.6 (1.3−8.5)	4.8 (1.3−10.5)	6.5 (2.2−12.2)	4.5 (1.5−10.0)
Died during follow‐up	25,552				
*n* (%)		1019 (8.2)	1323 (18.1)	763 (13.3)	3105 (12.2)

Abbreviations: IQR, interquartile range; MSM, men who have sex with men.

^a^ MSM (*n* = 11,578), males, other than MSM (*n* = 6558), females (*n* = 5315), overall (*n* = 23,451).

^b^ Males, other than MSM (*n* = 6832), females (*n* = 5305), overall (*n* = 24,626). Individuals are classified into only one risk group.

^c^ The category other includes individuals with a history of injection drug use, perinatal HIV infection, blood product transfusion or unspecified sexual exposure, as well as bisexual women, and those with risk defined as other.

During the follow‐up period, there were 27 incident cervical cancer diagnoses and 56 incident anal cancer diagnoses (40 MSM, 9 females and 7 males other than MSM). The median age at cervical cancer diagnosis was 34.3 years (IQR 29.7−44.3), occurring at a median of 2.3 years (IQR 1.2−6.9) after clinic entry. The median age at anal cancer diagnosis was 44.7 years (IQR 36.7−54.7), occurring a median of 6.8 years (IQR 2.9−9.5) after cohort entry. The median age at anal cancer diagnosis was 41.5 years (IQR 34.7−49.3) among MSM (40 events), 55.4 years (IQR 46.6−62.5) for females (nine events) and 52.7 years (IQR 50.3−54.3) for males other than MSM (seven events).

### 3.2 Incidence rates and temporal trends

**Figure 1:**
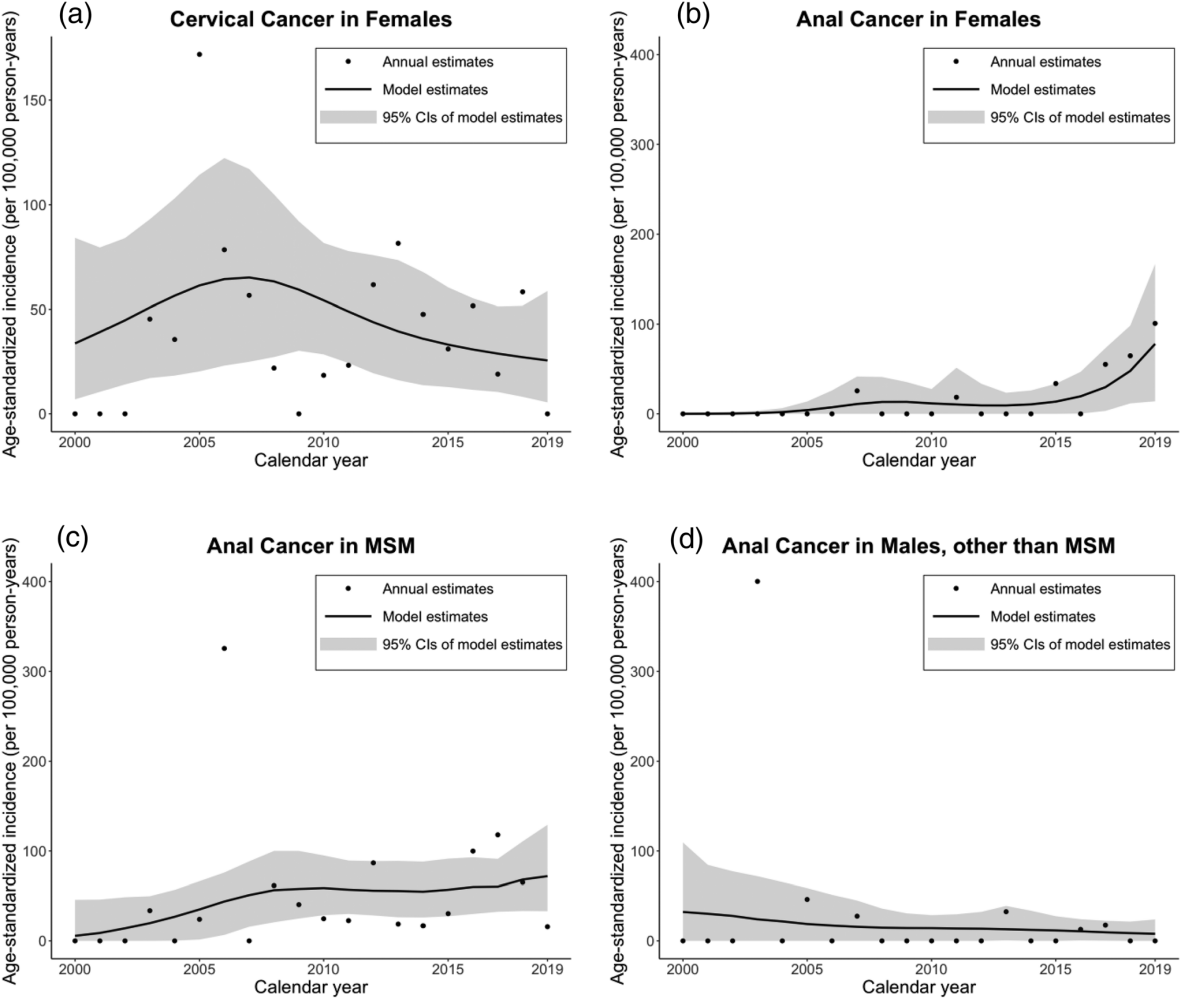
Annual age‐standardized incidence rates and age‐standardized Poisson model estimates, 2000–2019. 95% confidence interval based on bootstrap percentiles with 1000 replications. (a) Incidence rates of cervical cancer. (b) Incidence rates of anal cancer for females. (c) Incidence rates of anal cancer for MSM. (d) Incidence rates of anal cancer for males other than MSM. NB: The scales of the y‐axes differ between Figure 1a) and those of Figures 1b–d). Abbreviations: CI, confidence interval; MSM, men who have sex with men.

The overall crude incidence rate of cervical cancer was 62.2 per 100,000 person‐years (95% confidence interval [CI]: 34.9−89.4). For anal cancer, the crude incidence rates were 59.1 (95% CI: 33.2−85.0), 20.7 (95% CI: 11.6−29.7) and 15.2 (95% CI: 8.6−21.9) per 100,000 person‐years in MSM, females and males other than MSM, respectively. The number of cancer cases and person‐time per year is included in Table S1. The age‐standardized incidence rate of cervical cancer did not significantly change over the 20‐year period (*p* = 0.46) (Figure 1a). Although the incidence of anal cancer appears to have increased over time, especially in females and MSM (Figures 1b−d), the overall trend was not statistically different from no change (*p* = 0.25). In the Poisson regression model evaluating anal cancer incidence by sex/sexual HIV acquisition risk group, there were significant differences in age‐standardized incidence rates by group, with MSM having the highest rate (*p*<0.001). This difference did not change significantly over calendar years (interaction term *p* = 0.28). In analyses restricted to cancer diagnosis >90 days from enrollment (*n* = 54 anal cancers and *n* = 24 cervical cancers) among individuals who were followed for >90 days, trends were similar to the main analysis (Figure S3).

### 3.3 Characteristics associated with risks of anal and cervical cancer

In propensity score‐adjusted Cox proportional hazards models shown in Table 2, we examined factors associated with cancer risk, including year of clinic entry, time‐updated CD4 count, time‐updated ART use and sex/sexual HIV acquisition risk group (for anal cancer). There was no significant difference in the risk of anal cancer (*p* = 0.76) or cervical cancer (*p* = 0.41) by calendar year of clinic entry. Anal cancer risk decreased significantly with higher CD4 cell count (HR 0.65 [95% CI: 0.58−0.74] for CD4 400 vs. 200, HR 0.50 [95% CI: 0.44−0.55] for CD4 500 vs. 200). A similar trend was seen in the risk of cervical cancer, though this was not significant. For anal cancer, females (HR 0.34 [95% CI: 0.16−0.73]) and males other than MSM (HR 0.26 [95% CI: 0.11−0.62]) had significantly lower risks of anal cancer than MSM.

**Table 2:** Adjusted hazard ratios of anal and cervical cancer based on Cox proportional hazard models with propensity score adjustment for other covariates, stratification by clinical site and age as time scale

	Cervical cancer (*n* = 27)		Anal cancer (*n* = 56)	
	Adjusted HR [95% CI]	*p* value	Adjusted HR [95% CI]	*p* value
Calendar year of clinic entry		0.409		0.757
2010	Reference		Reference	
2005	1.78 [0.78−4.05]		1.12 [0.85−1.49]	
2015	1.03 [0.66−1.60]		1.34 [1.14−1.57]	
2019	1.41 [0.21, 9.74]		2.06 [0.90−4.73]	
Time‐updated CD4 cell count (cells/µl)		0.199		0.010
100	1.46 [0.17−12.2]		1.04 [0.5−2.14]	
200	Reference		Reference	
300	0.78 [0.57−1.07]		0.84 [0.76−0.93]	
400	0.65 [0.44−0.95]		0.65 [0.58−0.74]	
500	0.57 [0.41−0.78]		0.50 [0.44−0.55]	
Time‐updated ART use		0.527		0.784
Never	Reference		Reference	
Ever	1.92 [0.25−14.51]		1.20 [0.35−4.1]	
Sex/sexual HIV acquisition risk group	N/A	N/A		0.001
MSM			Reference	
Females			0.34 [0.16−0.73]	
Males, other than MSM			0.26 [0.11−0.62]	

Abbreviations: ART, antiretroviral therapy; CI, confidence interval; HR, hazard ratio; MSM, men who have sex with men; N/A, not applicable.

### 3.4 Mortality after anal and cervical cancer diagnosis

**Figure 2:**
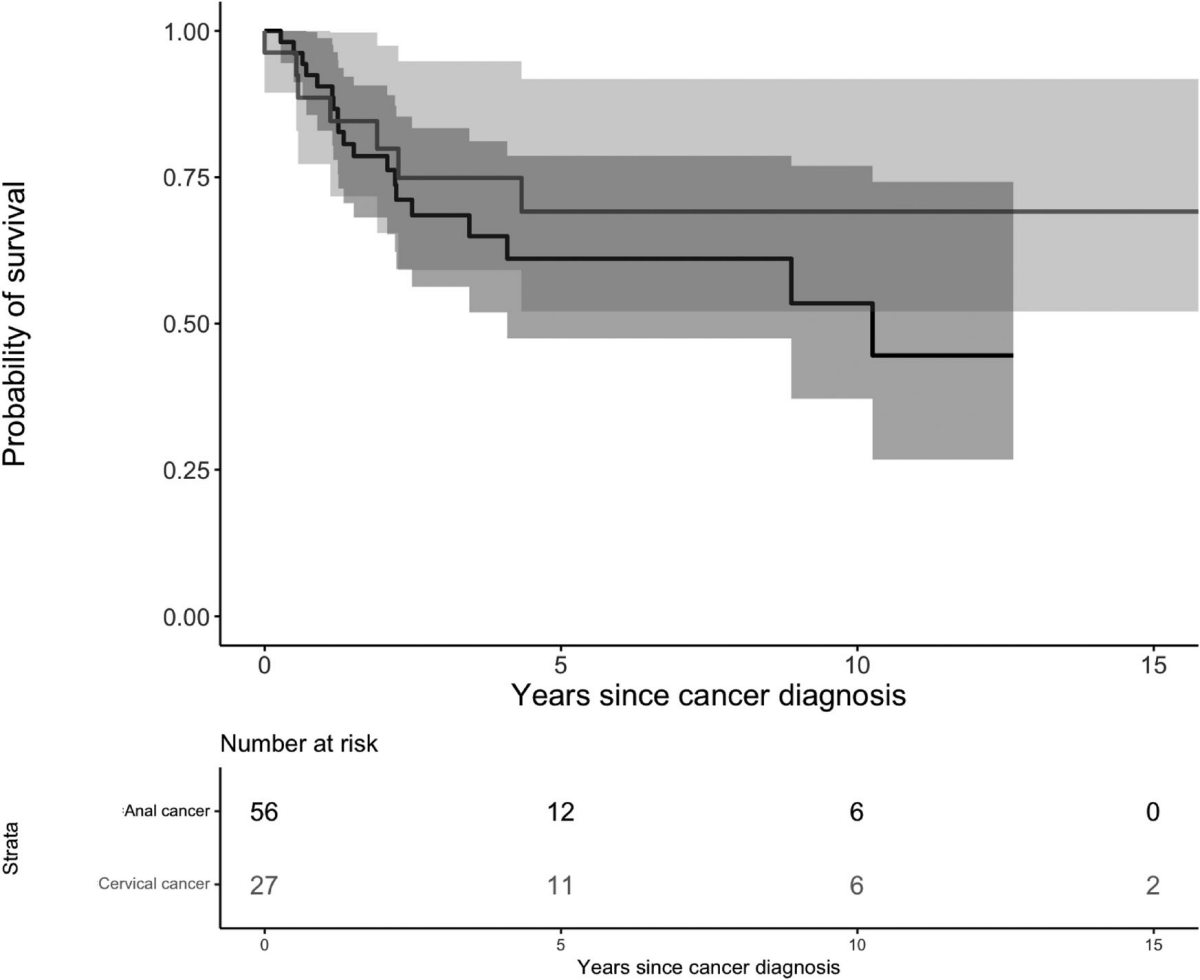
Kaplan−Meier curve of survival after cervical cancer (light grey line) and anal cancer (black line) diagnosis.

Among individuals diagnosed with anal or cervical cancer, we examined the mortality risk following cancer diagnosis (Figure 2). Of the 27 females with incident cervical cancer, seven died during follow‐up. The predicted probability of 5‐year survival was 72.6% (95% CI: 48.4−86.8). Of the 56 PWH with incident anal cancer, 24 died during follow‐up (16/40 MSM, 4/9 females and 4/7 males other than MSM) with an overall predicted probability of 5‐year survival 58.5% (95% CI: 44.0−70.5).

### 3.5 Cancer screening and HPV vaccination

**Figure 3:**
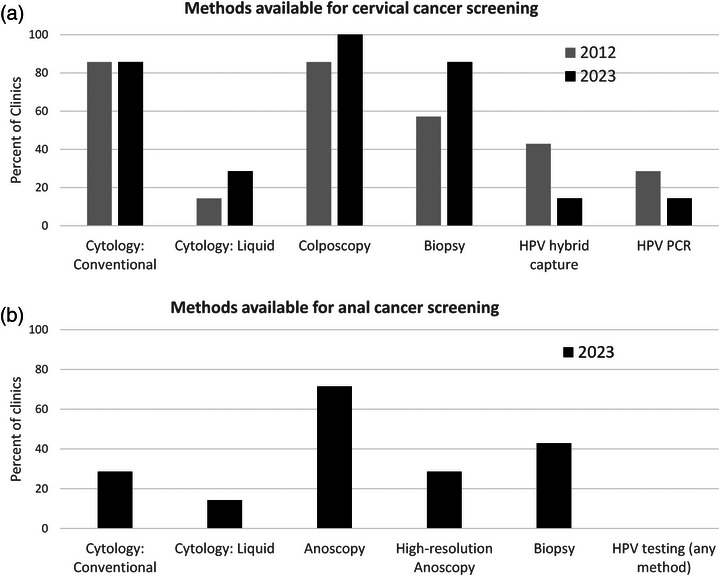
Available methods for cancer screening. (a) Methods available for cervical cancer screening at seven CCASAnet sites in 2012 and 2023. (b) Methods available for anal cancer screening at seven CCASAnet sites in 2023. Questions regarding anal cancer screening methods were added in 2023. Abbreviations: HPV, human papillomavirus; PCR, polymerase chain reaction.

Seven CCASAnet sites participated in both the 2012 and 2023 surveys of screening practices. In both years, all sites reported having cervical cancer screening availability for patients either on site (where HIV care is provided) or off site, such as through a community clinic or a referral to a private clinic. Each screening method was asked about individually, and cytology (conventional or liquid‐based) was identified as an initial cervical cancer screening method in all clinics in 2023. Two sites routinely offered HPV testing (PCR or hybrid capture) in 2023, compared to three sites offering any availability in 2012 (Figure 3a). Anal cancer screening availability for males increased from 3/7 to 4/7 sites from 2012 to 2023, and 3/7 sites offered anal cancer screening for women in both 2012 and 2023. In 2023, the most frequent reason for anal cancer screening was clinical suspicion for malignancy (4/7), followed by asymptomatic screening of MSM (3/7). Currently, anoscopy with visual inspection is the most available anal cancer screening method (5/7), with two of these sites also offering high‐resolution anoscopy. All anoscopies are performed by colorectal surgeons or gastroenterologists. Few (3/7) sites offer anal cytology (Figure 3b), and no sites reported national guidelines for anal cancer screening. Most sites reported recommending HPV vaccination for adolescent females (6/7) or adolescent males (5/7), but only three sites reported vaccination for adult PWH.

## 4 DISCUSSION

In this multinational cohort analysis of incident cervical and anal cancer in PWH in Latin America, we found no statistically significant change in the incidence of either malignancy from 2000 through 2019. Cervical cancer incidence remains high in the region, with a younger median age of diagnosis compared to anal cancer. The risk of developing anal cancer was higher among MSM when compared to other PWH and decreased with increasing time‐updated CD4 count. Screening for cervical cancer by cytology remains widely available to PWH at CCASAnet sites, while HPV testing availability is limited. Sites report fewer resources and differing practices for anal cancer screening.

While the incidence of anal cancer in PWH is documented in Europe and the United States, our study is one of the first to report anal cancer incidence in Latin America in PWH [4, 16, 21]. We found stable (or perhaps increasing, though not statistically significant) age‐standardized rates of anal cancer over the 20‐year period, while the trends of anal cancer in PWH in the United States and Europe have been stable or declining in recent years [22, 23, 24]. Mortality after anal or cervical cancer diagnosis was similar to published rates from U.S. cohorts [25]. The difference in these regional trends is likely multifactorial. Lower CD4 count as well as increasing age have been associated with higher incidence of anal cancer, but the risk from older age due to increased survival in the post‐ART era may be balanced or outweighed by improved immune status [4, 23, 26]. The growing availability of HPV vaccination and anal cancer screening is likely to have an increasing influence on regional incidence rates as these measures are implemented globally.

Anal cancer screening and treatment of anal pre‐cancerous lesions is now recommended starting at age 35 for MSM with HIV and at age 45 for other individuals with HIV [11, 12]. The median age at anal cancer diagnosis for both MSM and other PWH in our cohort was higher than these newly recommended ages of screening, though 11 individuals (10 MSM and 1 female) were diagnosed before reaching their recommended screening age. The most common group offered asymptomatic screening at CCASAnet sites was MSM, a strategy supported by findings in our study. One concern with expanding the use of cytology for screening is that abnormal results should be followed by evaluation with high‐resolution anoscopy, which requires specialized training and equipment [12]. In our study, most sites reported only access to anoscopy through referral to a specialist, which may contribute to delays in diagnoses and access to treatment.

For cervical cancer, published age‐standardized incidence rates from 2020 in Central and South America were 13.8 and 15.4 per 100,000 person‐years, respectively, still well above the target incidence of 4 cases per 100,000 person‐years set by the WHO Cervical Cancer Elimination Initiative [15]. The crude incidence rate of cervical cancer among females with HIV in our cohort was notably much higher (62.2 per 100,000 person‐years) than those estimates. Additionally, the incidence was statistically stable over time, which contrasts with the decrease in cervical cancer incidence seen in this region from 1990 to 2019 [27]. Cervical cancer risk also generally increases with age, yet the median age at diagnosis in our cohort was 34.3 years, with diagnoses occurring in the first few years after clinic entry [3]. The cancer screening history of individuals in our cohort before and after HIV clinic entry is unknown, but likely influences the median age at diagnosis.

Cervical cancer screening and treatment of pre‐cancerous lesions are two components of the WHO Cervical Cancer Elimination Strategy [10]. All CCASAnet sites surveyed reported national cervical cancer screening guidelines with varying recommendations [28]. All sites reported access to cervical cancer screening either on or off site with cytology for individuals enrolled in HIV care. Access to HPV testing, which is the WHO recommended screening method, remains limited in our cohort, as in much of the world [14]. These data support continued efforts to expand gender‐neutral HPV vaccination before the initiation of sexual activity and access for young adults at high risk for HPV, including PWH.

There are several limitations that should be considered. CCASAnet includes many tertiary referral clinics, and thus, the patient populations, counselling services and cancer prevention services may not be representative of sites in each country. Additionally, the CCASAnet sites represent a diversity of countries and healthcare systems. While we were able to verify each case of cervical and anal cancer, we did not have consistent data on previous cancer screening, pre‐cancerous lesions, HPV co‐infection, HPV vaccination status, cancer stage or complete cancer treatment data, so we were unable to include these factors in the analyses. By using all cancer diagnoses after clinic enrollment, some prevalent cancers may have been included in our analysis, but we sought to account for this with sensitivity analyses. We did not have the ability to exclude those with prior hysterectomy from the cervical cancer analysis or to include gender identity in either analysis. Though the overall cohort is large, more extensive investigation, including assessing incidence in subgroups, is limited by the small number of cases. The site surveys provide a helpful cross‐sectional view of cancer screening services in 2012 and 2023, but do not capture the full breadth of screening practices over the study period. Our analysis is limited to years before the COVID‐19 pandemic due to subsequent disruptions in data reporting. Decreased cancer screening during the early months of the pandemic was reported globally, including in South America, which likely impacted cancer diagnoses during this period [29, 30]. For example, cancer incidence in the United States declined in 2020 and then returned to pre‐pandemic rates in 2021 [31]. Further studies are needed to assess the effect of the COVID‐19 pandemic on cancer screening practices and cancer incidence in PWH in Latin America.

## 5 CONCLUSIONS

In one of the few reports outside the United States or Europe, we observed age‐standardized cervical and anal cancer incidence rates in our multinational cohort of PWH to be fairly stable over the 20‐year study period. Higher CD4 count was associated with decreased risk of anal cancer, with a similar trend in cervical cancer, reinforcing the need for timely HIV diagnosis and treatment. As seen in other cohorts, we found higher anal cancer incidence in MSM compared to other PWH in our cohort, supporting the benefit of screening in this population. These data support continued efforts to expand access to gender‐neutral HPV vaccination and to evaluate optimal implementation strategies for cervical and anal cancer screening.

## COMPETING INTERESTS

VF declares participation as a speaker and member of an experts’ discussion for MSD. All other authors declare no competing interests.

## AUTHOR CONTRIBUTIONS

Data curation and resources by RV‐B, EMJ, BC, CPC, DV, GH, CR, EG, VF and JLC. Conceptualization and methodology by RAP, JLC, RV, EMJ, SLS, BC‐R, CPC, DV, EG. and VF. Investigation and formal analysis by ST, BES, RAP and JLC. Writing—original draft by RAP, ST and JLC. Funding acquisition by JLC and RAP and supervision by JLC. All authors contributed to the reviewing and editing of the manuscript. All authors had full access to the data in the study and accept responsibility for the decision to submit for publication.

## FUNDING

This work was supported by the NIH‐funded Caribbean, Central and South America network for HIV epidemiology (CCASAnet), a member cohort of the International epidemiology Databases to Evaluate AIDS (leDEA) (U01AI069923). This award is funded by the following institutes: National Institute of Allergy and Infectious Diseases (NIAID), *Eunice Kennedy Shriver* National Institute of Child Health & Human Development (NICHD), National Heart, Lung, and Blood Institute (NHLBI), National Institute of Diabetes and Digestive and Kidney Diseases (NIDDK), National Institute on Drug Abuse (NIDA), National Institute on Alcohol Abuse and Alcoholism (NIAAA), Fogarty International Center (FIC) and National Cancer Institute (NCI). RAP received funding through the Vanderbilt Infection Pathogenesis and Epidemiology Research (5T32AI007474‐29) and 5K12CA090625‐25. REDCap is funded through UL1 TR000445 from NCATS/NIH.

## DISCLAIMER

The content is solely the responsibility of the authors and does not necessarily represent the official views of the National Institutes of Health. This manuscript is the result of funding in whole or in part by the NIH. It is subject to the NIH Public Access Policy. Through acceptance of this federal funding, NIH has been given the right to make this manuscript publicly available in PubMed Central upon the Official Date of Publication, as defined by NIH.

## Supporting information




**Figure S1**: Cohort flowchart applying exclusion criteria.
**Figure S2**: The time‐updated median CD4 cell count of the cohort (right y‐axis) and the time‐updated percentage of the cohort that has started ART (left y‐axis) by calendar year.
**Table S1**: Number of cases of cervical cancer and person‐time under observation per year, and the number of cases of anal cancer and person‐time in each sex/sexual HIV acquisition risk group by calendar year.
**Figure S3**: Annual age‐standardized incidence rates and age‐standardized Poisson model estimates, 2000–2019, restricted to cancer diagnosis >90 days from enrollment among individuals who were followed for >90 days (*n* = 54 anal cancers and *n* = 24 cervical cancers). 95% confidence interval based on bootstrap percentiles with 1000 replications. (a) Incidence rates of cervical cancer. (b) Incidence rates of anal cancer for females. (c) Incidence rates of anal cancer for MSM. (d) Incidence rates of anal cancer for males other than MSM. NB: The scales of the y‐axes differ between Figure 1a and those of Figures 1b−d. CI, confidence interval; MSM, men who have sex with men.

## Data Availability

CCASAnet welcomes interested investigators to collaborate with us for the use of our deidentified, patient‐level data following research proposal review and approval by contributing sites. A data dictionary defining each field in the dataset is available upon request. Please visit www.ccasanet.org for additional instructions.
