# Supplementary material for: Incidence and outcomes of anal and cervical cancer among adults with HIV in Latin America: a retrospective cohort study
Source: J Int AIDS Soc. 2025 Oct 11;28(10):e70050. doi: 10.1002/jia2.70050 (PMC12514557; doi:10.1002/jia2.70050)
Supplement: Supplementary file 1 — Figure S1: Cohort flowchart applying exclusion criteria. Figure S2: The time‐updated median CD4 cell count of the cohort (right y‐axis) and the time‐updated percentage of the cohort that has started ART (left y‐axis) by calendar year. Table S1: Number of cases of cervical cancer and person‐time under observation per year, and the number of cases of anal cancer and person‐time in each sex/sexual HIV acquisition risk group by calendar year. Figure S3: Annual age‐standardized incidence rates and age‐standardized Poisson model estimates, 2000–2019, restricted to cancer diagnosis >90 days from enrollment among individuals who were followed for >90 days (n = 54 anal cancers and n = 24 cervical cancers). 95% confidence interval based on bootstrap percentiles with 1000 replications. (a) Incidence rates of cervical cancer. (b) Incidence rates of anal cancer for females. (c) Incidence rates of anal cancer for MSM. (d) Incidence rates of anal cancer for males other than MSM. NB: The scales of the y‐axes differ between Figure 1a and those of Figures 1b−d. CI, confidence interval; MSM, men who have sex with men. [file JIA2-28-e70050-s001.pdf]

Supplementary Material

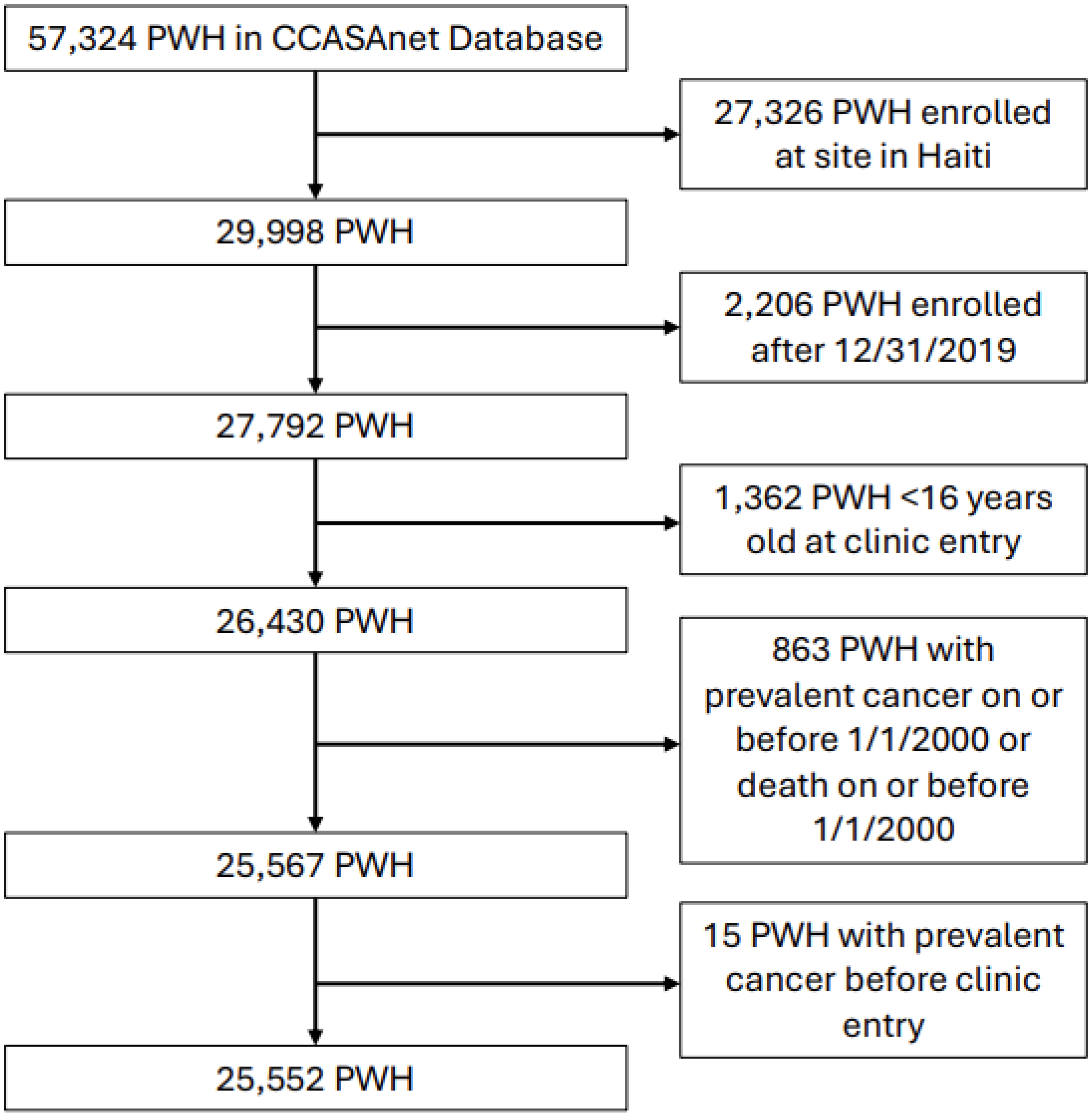

Supplementary Figure 1: Cohort flowchart applying exclusion criteria

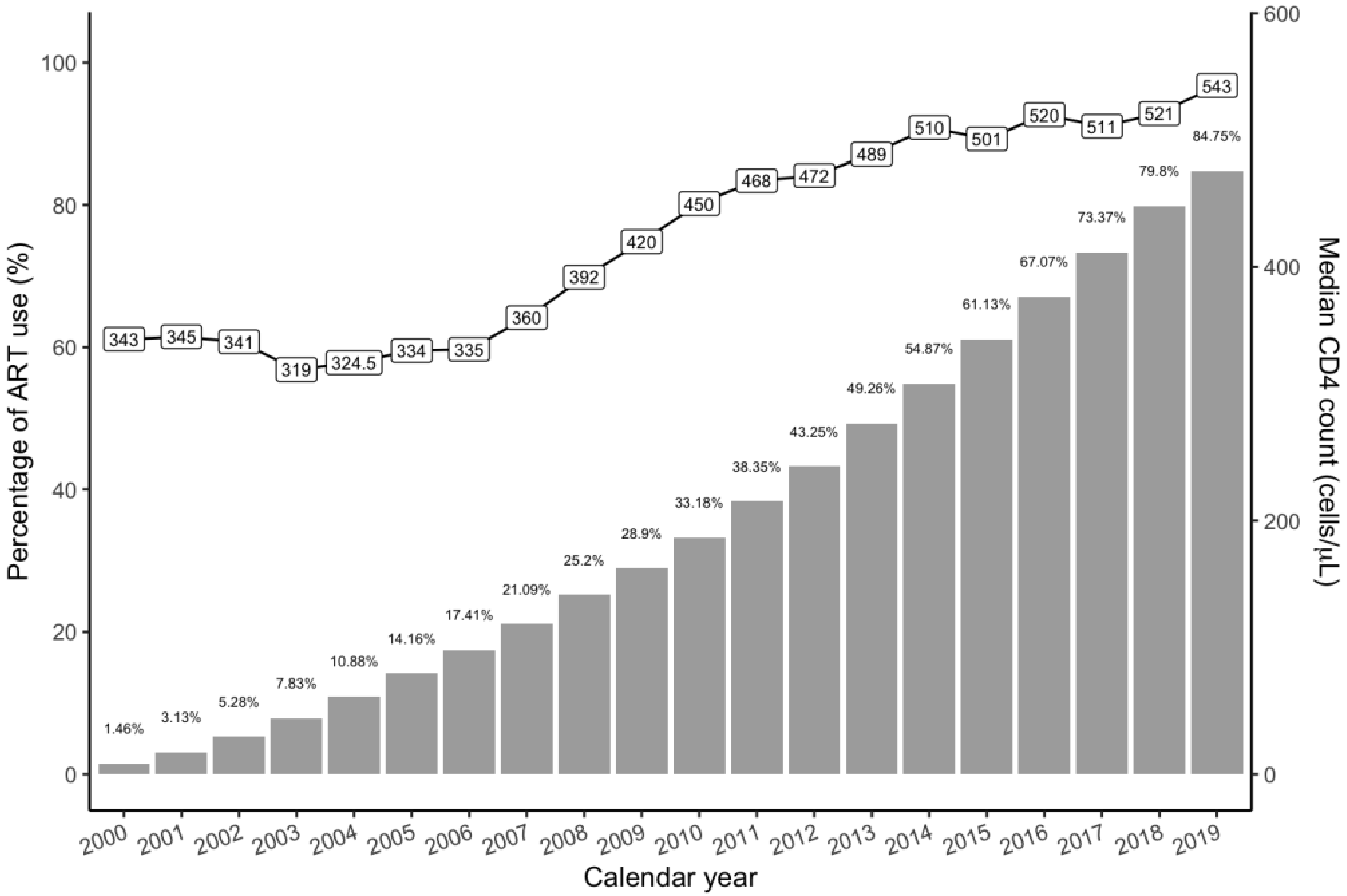

Supplementary Figure 2: The time-updated median CD4 cell count of the cohort (right y-axis) and the time-updated percentage of the cohort that has started ART (left y-axis) by calendar year.

Supplementary Table 1: Number of cases of cervical cancer and person-time under observation per year and the number of cases of anal cancer and person-time in each sex/sexual HIV acquisition risk group by calendar year.

	Anal cancer in women		Anal cancer in Males other than MSM		Anal cancer in MSM		Cervical cancer in women	
Years	Number of cases	Person years	Number of cases	Person years	Number of cases	Person years	Number of cases	Person years
2000	0	494.38	0	342.34	0	518.58	0	494.38
2001	0	656.16	0	521.46	0	722.32	0	656.16
2002	0	791.82	0	698.21	0	922.05	0	791.82
2003	0	930.10	1	856.35	1	1153.81	1	929.39
2004	0	1142.05	0	1086.71	0	1454.86	1	1140.24
2005	0	1363.97	1	1331.41	1	1767.52	3	1360.34
2006	0	1617.17	0	1619.67	1	2112.16	3	1611.34
2007	1	1857.48	1	1907.77	0	2419.04	2	1850.47
2008	0	2073.61	0	2183.12	3	2723.80	1	2066.25
2009	0	2287.50	0	2375.07	3	3041.79	0	2280.05
2010	0	2443.71	0	2541.73	2	3362.23	1	2436.63
2011	1	2650.26	0	2765.48	2	3746.62	1	2642.54
2012	0	2829.25	0	2980.79	2	4197.48	2	2820.09
2013	0	3023.53	2	3204.41	2	4738.42	2	3014.07
2014	0	3190.95	0	3403.91	2	5319.25	3	3179.31
2015	1	3288.40	0	3583.43	3	5825.51	2	3275.31
2016	0	3249.67	1	3596.90	4	5811.65	2	3238.63
2017	2	3221.62	1	3623.02	7	5548.89	1	3211.39
2018	2	3264.50	0	3691.02	5	5996.51	2	3255.15
2019	2	3169.19	0	3624.43	2	6301.46	0	3163.32

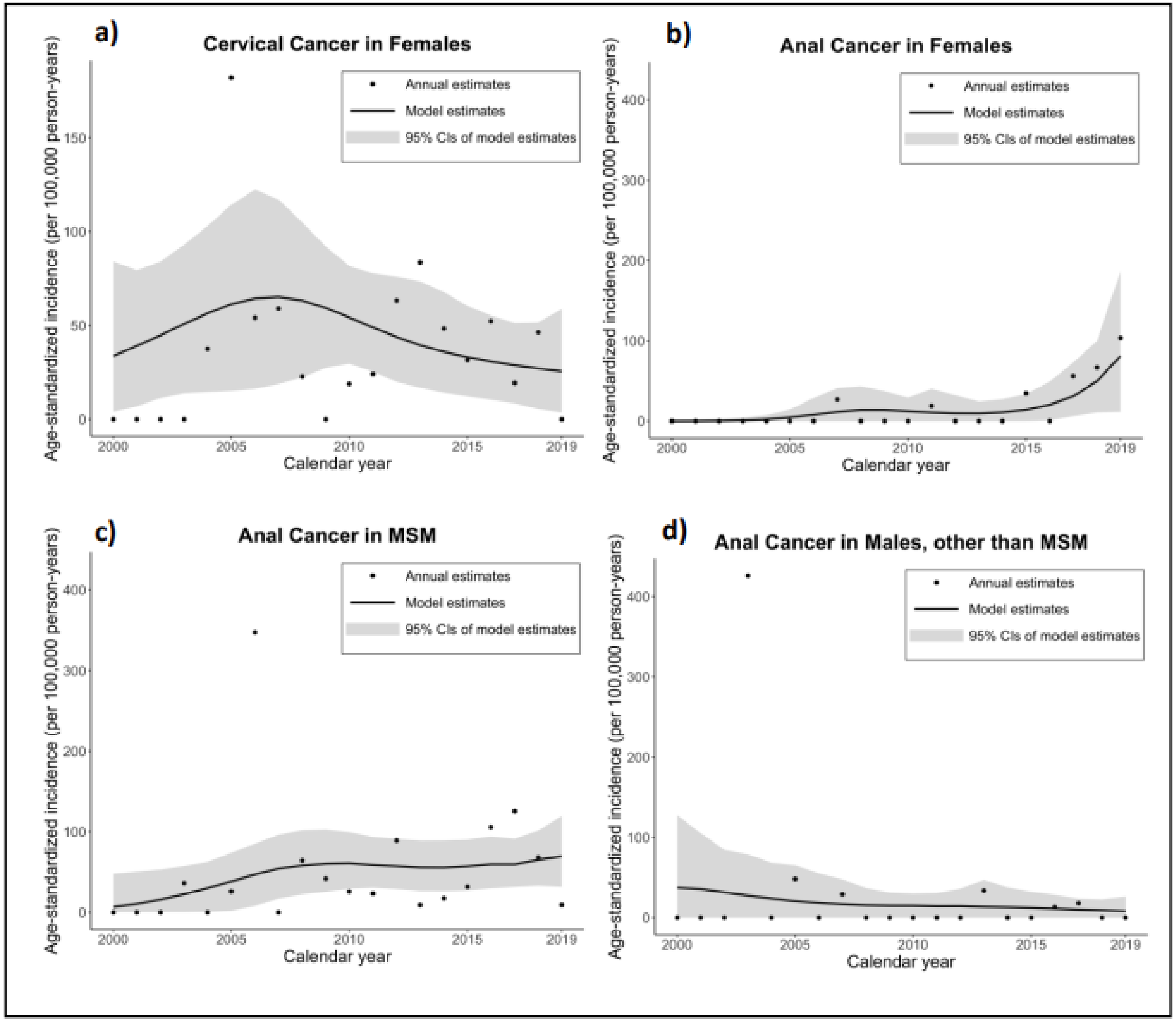

Supplementary Figure 3: Annual age-standardized incidence rates and age-standardized Poisson model estimates, 2000-2019, restricted to cancer diagnosis >90 days from enrollment among individuals who were followed for >90 days (n=54 anal cancers and n=24 cervical cancers). 95% confidence interval based on bootstrap percentiles with 1000 replications. (a) Incidence rates of cervical cancer. (b) Incidence rates of anal cancer for females. (c) Incidence rates of anal cancer for MSM. (d) Incidence rates of anal cancer for males other than MSM. NB: The scales of the y-axes differ between Figure 1a and that of Figures 1b, 1c and 1d.

CI: Confidence Interval, MSM: Men who have sex with men
